# Role of CD8^+^ T cell exhaustion in the progression and prognosis of acute respiratory distress syndrome induced by sepsis: a prospective observational study

**DOI:** 10.1186/s12873-022-00733-2

**Published:** 2022-11-19

**Authors:** Lei Yan, Yumei Chen, Yi Han, Chaoyang Tong

**Affiliations:** Department of Emergency Medicine, Zhongshan Hospital, Fudan University, Shanghai, 200032 China

**Keywords:** Sepsis, Acute respiratory distress syndrome, CD8^+^ T cell exhaustion, Coinhibitory receptors, Prognosis

## Abstract

**Background:**

CD8^+^ T cells are important for protective immunity against intracellular pathogens. Excessive amounts of antigen and/or inflammatory signals often lead to the gradual deterioration of CD8^+^ T cell function, a state called “exhaustion”. However, the association between CD8^+^ T cell exhaustion and acute respiratory distress syndrome (ARDS) has not been studied. This study was conducted to elucidate how CD8^+^ T cells and inhibitory receptors were related to the clinical prognosis of ARDS.

**Methods:**

A prospective observational study in an emergency department enrolled patients who were diagnosed with sepsis-associated ARDS according to the sepsis-3 criteria and Berlin definition. Peripheral blood samples were collected within 24 h post recruitment. CD8^+^ T cell count, proliferation ratio, cytokine secretion, and the expression of coinhibitory receptors were assayed.

**Results:**

Sixty-two patients with ARDS met the inclusion criteria. CD8^+^ T cell counts and proliferation rates were dramatically decreased in non-surviving ARDS patients. Increasing programmed cell death 1 (PD-1) expression on the CD8^+^ T cell surface was seen in patients with worse organ function, while an increasing level of T cell immunoglobulin mucin-3 (Tim-3) was associated with a longer duration of the shock. Kaplan–Meier analysis showed that low CD8^+^ T cell percentages and increased inhibitory molecule expression were significantly associated with a worse survival rate.

**Conclusions:**

CD8^+^ T cells and coinhibitory receptors are promising independent prognostic markers of sepsis-induced ARDS, and increased CD8^+^ T cell exhaustion is significantly correlated with poor prognosis.

**Supplementary Information:**

The online version contains supplementary material available at 10.1186/s12873-022-00733-2.

## Introduction

Sepsis is a serious clinical condition characterized by an unbalanced host’s inflammatory response to infection [1–3]. Due to excessive and uncontrolled inflammatory reactions, patients with sepsis always develop acute respiratory distress syndrome (ARDS), life-threatening disease with a high mortality rate [4, 5]. This syndrome is an inflammatory lung disorder caused by injury to the alveolar-capillary membrane, which results in increased alveolar-capillary permeability and protein-rich pulmonary edema. This leads to severe hypoxemia (assessed by the PaO2/FiO2 ratio < 300 mmHg), radiologic infiltrates, and decreased lung compliance [4].

In retrospective studies from China, about 30% of patients with sepsis developed ARDS, with a 28-day mortality rate as high as 47% [6–8]**.** In the setting of infectious lung injury, neutrophils accumulate in the lung microvasculature and release several toxic mediators, which result in a sustained loss of normal epithelial and endothelial barrier [9]. Lymphocytes play a role in the resolution of inflammation, the restoration of the epithelial and endothelial barrier, and the removal of protein-rich edema fluid [9, 10]. Therefore, lymphocyte dysfunction may play an important role in the progression of ARDS.

In clinical work, significant progressive lymphopenia and depletion of CD8^+^ T cells were associated with the severity of Covid-19 [11]. There is a possible correlation between CD8^+^ T cell exhaustion and the prognosis of ARDS [11–13]. Experiments have recently revealed that CD8^+^ T cells played a vital role in protecting against antigens and/or inflammatory signals in sepsis [14, 15]. High and persistent antigen and inflammatory stimulation during infection, on the other hand, may change CD8^+^ T cell differentiation or cause CD8^+^ T cell exhaustion [16, 17]. Exhausted CD8^+^ T cells are distinguished by the gradual decrease of functional activities (cytokine synthesis and secretion), as well as overexpression of several inhibitory receptors (such as programmed cell death 1 (PD-1) and T cell immunoglobulin mucin-3 (Tim-3)), dysregulated homeostatic proliferation [14, 18, 19]. The clinical consequences of CD8^+^ T cell exhaustion in the development and prognosis of sepsis-induced ARDS are still unknown.

Building on the clinical observations and the outcomes of previous studies, we hypothesized that mortality in ARDS patients would be associated with the possible deficiency of CD8^+^ T cells. Here, we performed a phenotypic, proliferation, and cytokine secretion study on CD8^+^ T cells isolated from the peripheral blood of ARDS patients. The primary objective of this prospective observational study was to determine the relationship between CD8^+^ T cell exhaustion and the prognosis of ARDS caused by sepsis.

## Methods

### Study design and patients’ enrollment

**Fig. 1:**
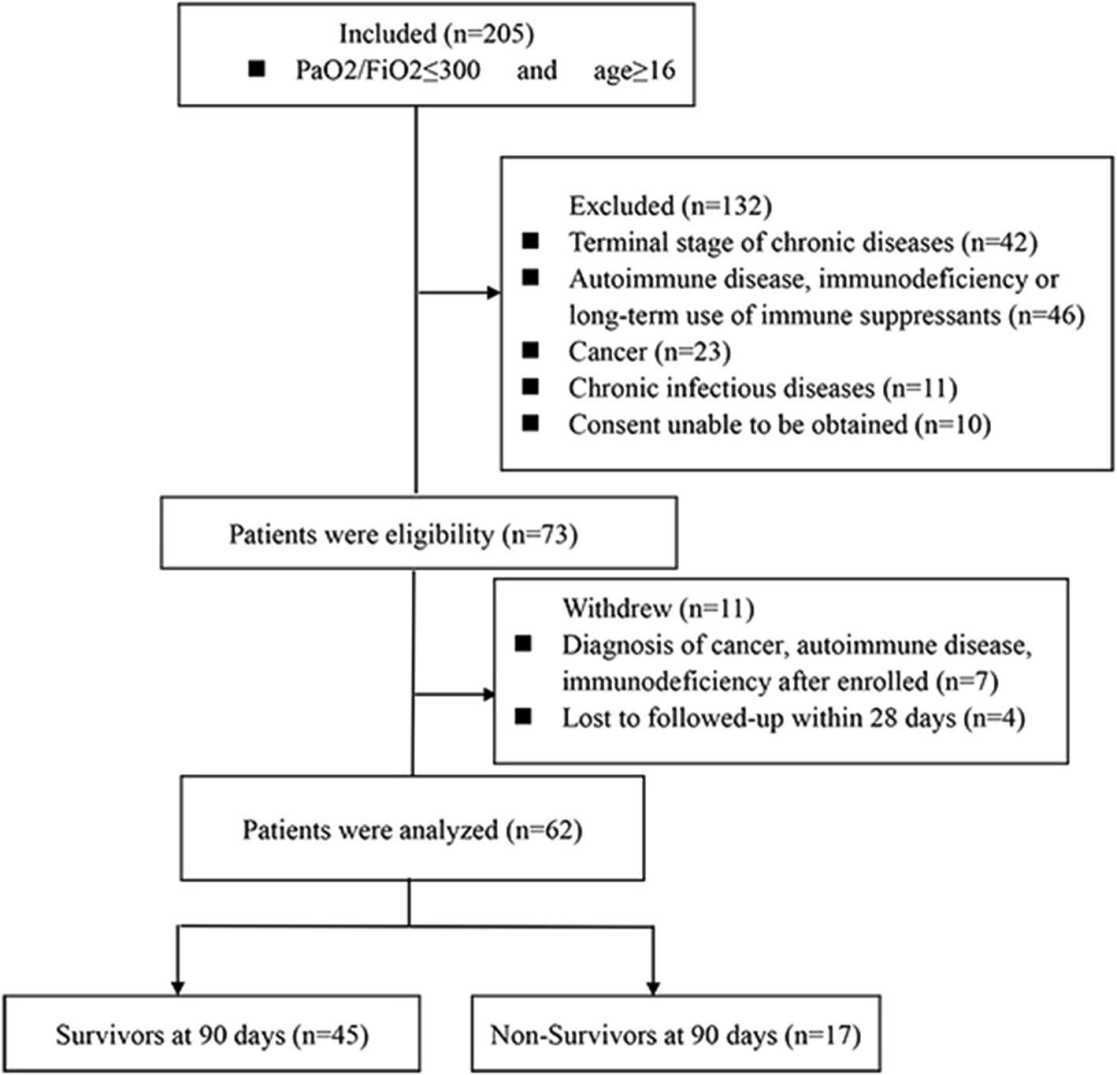
Patient enrollment flow diagram

Between December 2018 and June 2021, a prospective observational study was conducted by enrolling sepsis-associated ARDS patients (Fig. 1). According to the recommendations of the Surviving Sepsis Campaign in 2016 [2], this study included patients suspected of having an infection during emergency department admission (within 24 h) with a Sequential Organ Failure Assessment (SOFA) score of ≥2. We defined suspected sepsis as all of the following within 24 hours of hospital arrival: (1) a blood culture order (regardless of result), (2) Two or more SIRS criteria (body temperature more than 38 °C or less than 36 °C; heart rate more than 90/minute; respiratory rate more than 20/minute; or white blood cell count more than 12,000 cells/uL or less than 4000 cells/uL). We selected ARDS patients from those diagnosed with suspected sepsis according to the Berlin definition [4]. According to the Berlin diagnostic criteria of ALI/ARDS, ARDS was defined by the following parameters: (1) mechanical ventilation and positive end-expiratory pressure or continuous positive airway pressure ≥ 5 cmH2O;(2) acute onset of bilateral infiltrates on chest radiograph or CT; (3) severe (PaO2/FiO2 ≤ 100 mmHg), moderate (PaO2/FiO2 = 100–200 mmHg), or mild (PaO2/FiO2 = 200–300 mmHg); and (4) without pleural effusion, lung collapse, lung nodules, or cardiogenic pulmonary edema. Informed consent was obtained from all patients or their legal proxies before enrollment. This prospective observational study was conducted in our emergency department and approved by the institutional ethics committee.

Patients were excluded if any of the following criteria were fulfilled: younger than sixteen years old, end-stage chronic disease and estimated survival time < 28 days, autoimmune disease, immunodeficiency, tumor, chronic infectious diseases, received chemotherapy within six months, or consent could not be obtained.

### Data collection

Baseline characteristics, including demographic data, the site of infection, organ function, pathogenic microorganisms, disease severity, and prescribed drugs, were recorded within 24 h after satisfying the criteria for sepsis-related ARDS. The severity of the disease was assessed using the Acute Physiology and Chronic Health Evaluation II (APACHE II) [20], sequential organ failure assessment (SOFA) scores [21], Lung Injury Prediction Scores (LIPS), and shock duration. Shock duration was defined as the total number of days of hypotension requiring vasopressor support. The patients were followed for at least 90 days after enrollment. According to the mortality within 90 days, the patients were divided into non-survival and survival groups.

### Blood sampling and measurements

Within 24 h after the diagnosis of sepsis-associated ARDS, approximately 5 ml peripheral whole blood samples were collected in tubes containing heparin or ethylene diamine tetraacetate acid (EDTA). White blood cell count, C-reactive protein, and procalcitonin were immediately measured in the clinical chemistry laboratory of the hospital.

Peripheral blood mononuclear cells (PBMCs) were isolated via Ficoll-Hypaque density gradient centrifugation following standard protocols. The cells were washed and resuspended in T cell medium (Roswell Park Memorial Institute (RPMI) 1640 medium supplemented with 1% penicillin-streptomycin, 10% heat-inactivated fetal bovine serum, L-glutamine, and nonessential amino acids) and processed for flow cytometry, proliferation or cytokine secretion evaluations as described below.

### Flow cytometry

Antibodies specific to CD8 (MultiSciences, Hangzhou, Zhejiang, China), PD-1 (eBioscience, Ankara, TURKEY), and Tim-3 (BD Biosciences, San Jose, CA, USA) were used for surface staining of cells. All experiments were carried out with a CytoFLEX LX flow cytometer (BECKMAN COULTER, BREA, CA, USA), with a minimum of 1,000,000 events per sample. Calibrator beads were used to calibrate the FACS machine before each run. Cells were gated on live cells based on forward- and side-scatter properties. The samples were analyzed using FCS Express software version 7.0 (De Novo Software, Los Angeles, CA).

### CD8^+^ T cell isolation and proliferation ability test

CD8^+^ T cells were purified from human PBMCs according to an EasySep Human CD8^+^ T Cell Iso Kit (Stemcell Technologies, Vancouver, Canada) user manual. Flow cytometry showed that the CD8^+^ T cell purity was > 98%. For the Cell Counting Kit-8 assay, we employed a Cell Counting Kit (Yeasen, China) to determine the proliferation rate of cells. The optical absorbance was measured at 450 nm. All operation steps were carried out in accordance with the instructions of the kit manual.

### Cytokine analysis

The purified CD8^+^ T cells were cultured in 24-well plates and stimulated with phorbol-12-myristate-13-acetate (PMA). The culture supernatant was harvested at 24 h following stimulation, and cytokine levels were determined using ELISA kits (MultiSciences, Hangzhou, Zhejiang, China) according to the manufacturer’s instructions. We measured human Tumor necrosis factor-alpha (TNF-α) and Interferon-gamma (IFN-γ) levels in CD8^+^ T cells.

### Statistical analysis

Data were entered and analyzed using Statistical Package for Social Sciences version 21.0 (IBM, USA, New York) and GraphPad Prism version 7.0 (GraphPad Software, California, United States of America).

Values are presented as the means ± standard deviations when normality was confirmed and as medians with percentiles (25–75%) when the distribution was not normal. Categorical data are presented as numbers of events and percentages. For two-group comparisons, the independent samples t-test was used for normally distributed data, and the Mann–Whitney test was used for nonnormally distributed data. Categorical data were compared using the chi-square or Fisher’s exact test. Spearman’s rank correlation was applied to determine the correlation between variables.

The area under the receiver operating characteristic (ROC) curve was calculated to evaluate the prognostic value of the tested parameters. The optimal cutoff points of the percentage of lymphocytes, CD8^+^ T cells, PD-1^+^ CD8^+^ T cells, and Tim-3^+^ CD8^+^ T cells were assessed by the best Youden index (sensitivity + specificity-1) for mortality and secondary infection prediction, respectively. Kaplan–Meier plots and the log-rank test were used to compare survival and probability of secondary infection. Missing values had been included for survival analysis. All tests were two-tailed, and a value of *P* < 0.05 was considered statistically significant.

## Results

### Patient enrollment and characteristics

During the study period, a total of 205 patients were prospectively enrolled in this study (Fig. 1). Of these, 132 patients were excluded based on exclusion criteria and 11 patients withdrew from the study. Finally, 62 patients completed the study. Seventeen patients died within 90 days (Fig. 1).

The clinical characteristics of the patients are displayed in Table 1. There were no significant differences in sex, age, BMI, source of infection, or hormone usage between the survivors and non-survivors, and the *P* values were 0.81, 0.12, 0.24, 0.2, and 0.14, respectively. However, the differences in days of mechanical ventilation [4.00 (0-14.00) vs. 18.00 (7.00-39.00), *p* = 0.002] and septic shock [0 (0-3.50) vs. 4 (1-15.50), *p* < 0.001] between the two groups were conspicuous.

**Table 1:** Baseline characteristics of the patients according to 90-day survival

Parameters	All patients *n* = 62	Survivors *n* = 45	Non-survivors *n* = 17	*p*-Value
**Demographic characteristics**				
Female, n (%)	24 (38.71)	17 (37.78)	7 (41.18)	0.806
Age, y, mean (SD)	60.50 (15.16)	58.67 (14.92)	65.35 (15.14)	0.122
BMI, kg/m2, mean (SD)	24.61 (3.95)	24.97 (3.63)	23.63 (4.68)	0.236
**Severity of illness**				
Septic shock, median days (IQR)	1(0-6)	0(0-3.5)	4(1-15.5)	0.000
PaO2/FiO2 ratio, mean (SD)	212.56 (103.29)	234.36 (99.39)	154.88 (92.97)	0.006
APACHE II score, mean (SD)	12.23 (8.08)	10.40 (6.94)	17.06 (9.09)	0.003
SOFA score, median (IQR)	4.00(3.00-7.25)	4.00(2.50-5.50)	8.00(4.00-12.00)	0.001
LIPS score, mean (SD)	6.76 (3.05)	5.78 (2.90)	9.35 (1.60)	0.000
**Source of infection, n (%)**				
Pneumonia	45(72.58)	34(75.56)	11(64.71)	0.197
Non-pulmonary sepsis	17(27.42)	16(35.56)	1(5.88)	0.197
**Pathogenic microorganisms, n (%)**				
Gram-positive bacteria	5(8.06)	4(8.89)	1(5.88)	1.000
Gram-negative bacteria	33(53.23)	25(55.56)	8(47.06)	0.550
Virus	15(24.19)	11(24.44)	4(23.53)	1.000
Others	9(14.52)	5(11.11)	4(23.53)	0.404
**Inflammation indicators**				
C-reactive protein (mg/L), mean (SD)	72.17 (66.50)	63.56 (64.52)	94.95 (68.18)	0.098
Procalcitonin (ng/mL), median (IQR)	0.77(0.20-3.11)	0.61(0.11-4.92)	1.63(0.69-2.74)	0.124
**Prescribed drugs**				
Hormone	27(43.55)	17(37.78)	10(58.82)	0.136
Mechanical ventilation, median days (IQR)	7.00(0-18.25)	4.00(0-14.00)	18.00(7.00-39.00)	0.002
Hospital duration, mean days (SD)	26.19 (21.73)	24.20 (12.70)	31.47 (36.29)	0.430

*Abbreviations: BMI* Body Mass Index, *SD* Standard Deviation, *IQR* Interquartile Range, *APACHE* Acute Physiology and Chronic Evaluation, *SOFA* Sequential Organ Failure Assessment, *LIPS* Lung Injury Prediction Score

Non-survivors had higher APACHE II scores [17.06 ± 9.09 vs. 10.40 ± 6.94, *p* = 0.003] and SOFA scores [8.00 (4.00-12.00) vs. 4.00 (2.50-5.50), *p* = 0.001] than survivors. In addition, significantly lower PaO2/FiO2 levels [154.88 ± 92.97 VS. 234.36 ± 99.39, *p* = 0.006] and higher LIPS scores [9.35 ± 1.60 vs. 5.78 ± 2.90, *p* < 0.001] were observed among non-survivors.

### Decreased numbers of total lymphocytes and CD8^+^ subsets in sepsis-related ARDS patients

**Fig. 2:**
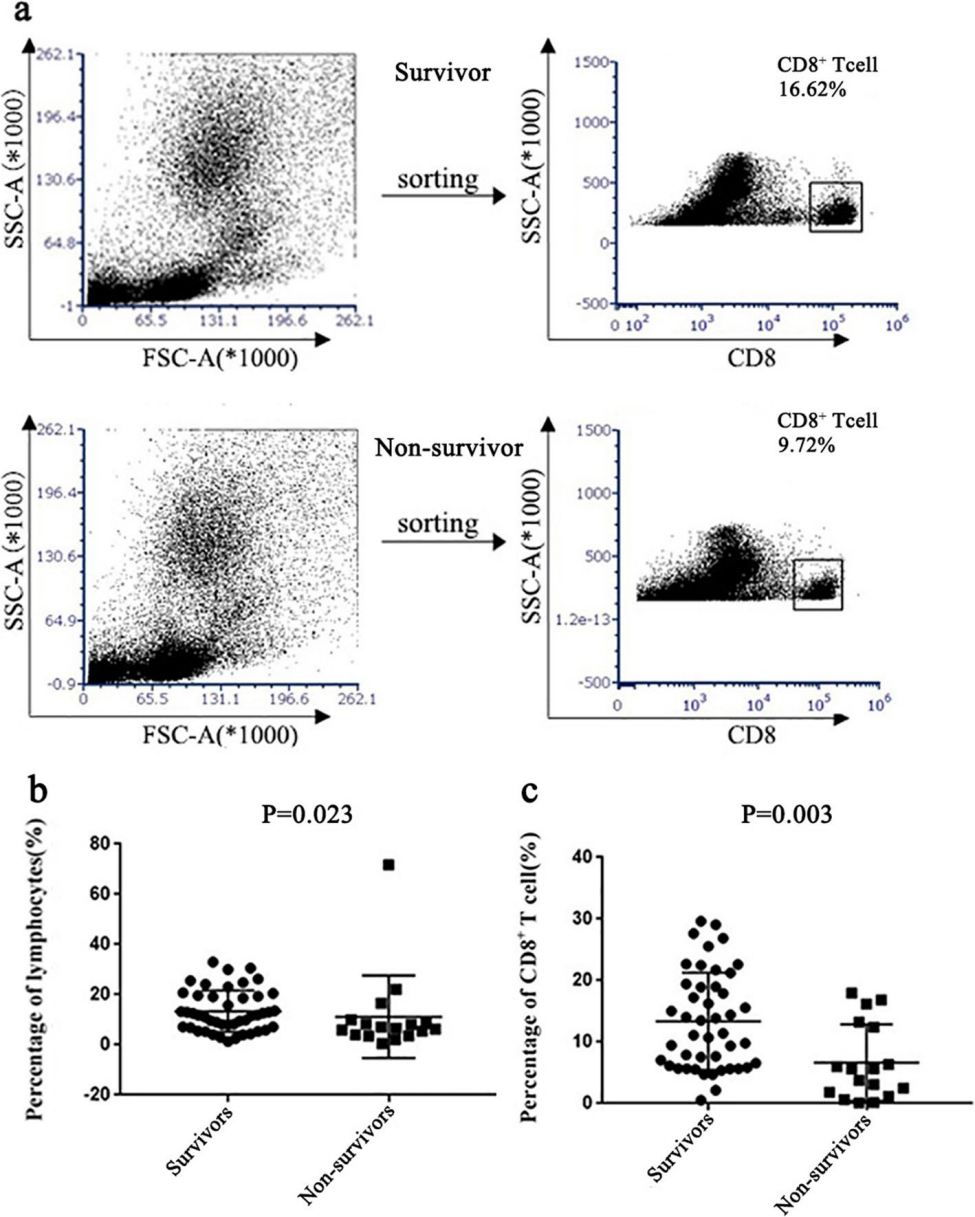
Percentage of lymphocytes and CD8^+^ T cells in ARDS patients. **a** Flow dot plots of the percentage of comparison CD8^+^ T cells between a non-survivor and a survivor. **b** and **c** Dot plots show the percentage of ARDS patients’ lymphocytes and CD8^+^ T cells to ascertain the significant differences between the non-survivor and survivor groups

As shown in Table 2 and Fig. 2, the absolute number and ratio of lymphocytes and CD8^+^ subsets in the peripheral blood of ARDS patients decreased more significantly in the non-survivor ARDS patients (*P* < 0.001). The proliferation ratio of CD8^+^ T cells of non-survivors was lower than that of survivors [154.88 ± 92.97 VS. 234.36 ± 99.39, *p* = 0.006].

**Table 2:** Comparison of exhaustion-related performance in patients between the survival and non-survival group

Parameters	All patients *n* = 62	Survivors *n* = 45	Non-survivors *n* = 17	p-Value
Lymphocytes percentage (%), median (IQR)	9.60(5.80-18.83)	11.80(7.05-19.55)	6.70(3.80-9.25)	0.023
Lymphocytes (×10^9/L), mean (SD)	1.11(0.65)	1.22(0.62)	0.83(0.65)	0.034
CD8^+^ T cell percentage (%), mean (SD)	11.53(8.00)	13.36(7.89)	6.68(6.19)	0.003
CD8^+^ T cell(×10^6/L), mean (SD)	216.15(188.97)	254.70(204.62)	106.00(55.16)	0.007
Cell proliferation ratio, mean (SD)	0.87(0.41)	1.01(0.38)	0.64(0.35)	0.045
IFN-γ (pg/ml), mean (SD)	14.46(13.69)	12.70(13.43)	20.65(13.95)	0.214
TNF-α (pg/ml), mean (SD)	7.88(7.08)	7.44(7.60)	9.39(5.12)	0.563
PD-1in CD8^+^ T cell (%), mean (SD)	15.46(14.88)	13.02(12.42)	21.93(18.93)	0.034
Tim-3 in CD8^+^ T cell (%), mean (SD)	19.03(16.24)	15.47(14.20)	28.43(17.94)	0.004

*Abbreviations: IQR* Interquartile Range, *SD* Standard Deviation, *TNF-α* Tumor necrosis factor-alpha, *IFN-γ* Interferon-gamma, *PD-1* Programmed Cell Death 1,*Tim-3* T-cell immunoglobulin mucin-3

### Expression characteristics of PD-1 and Tim-3 in peripheral CD8^+^ T cells during the onset of ARDS

**Fig. 3:**
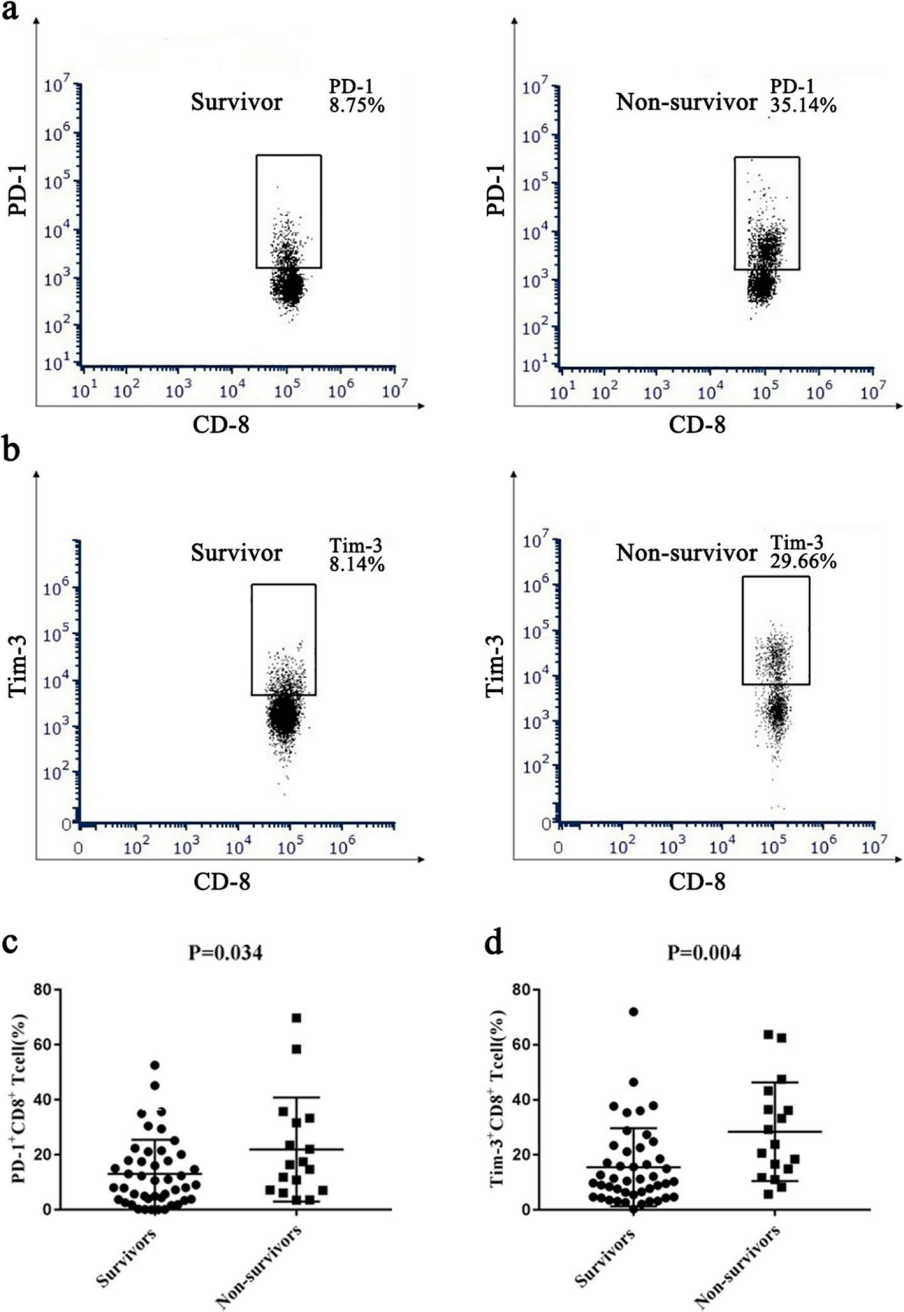
The expression of PD-1 and Tim-3 on CD8^+^ T cells in ARDS patients. **a** and **b** Representative flow cytometry images of PD-1 and Tim-3 on CD8^+^ T cells of a non-survivor and a survivor. **c** and **d** Dot plots show the percentage of PD-1^+^CD8^+^ T cells and Tim-3^+^CD8^+^ T cells of ARDS patients to ascertain the significant differences between the non-survivors and the survivors

In the patients who died, the expression of PD-1 and Tim-3 on CD8^+^ T cells was significantly elevated compared with that in survivors (*p* < 0.05) (Fig. 3). However, in intracellular cytokine analyses, IFN-γ and TNF-α expressed by CD8^+^ T cells were not obviously elevated in these two groups, and the *P* values were 0.21 and 0.56 (Table 2).

### Correlation of lymphocytes, CD8^+^ T cells, and co-signaling molecules with disease severity and outcome

**Fig. 4:**
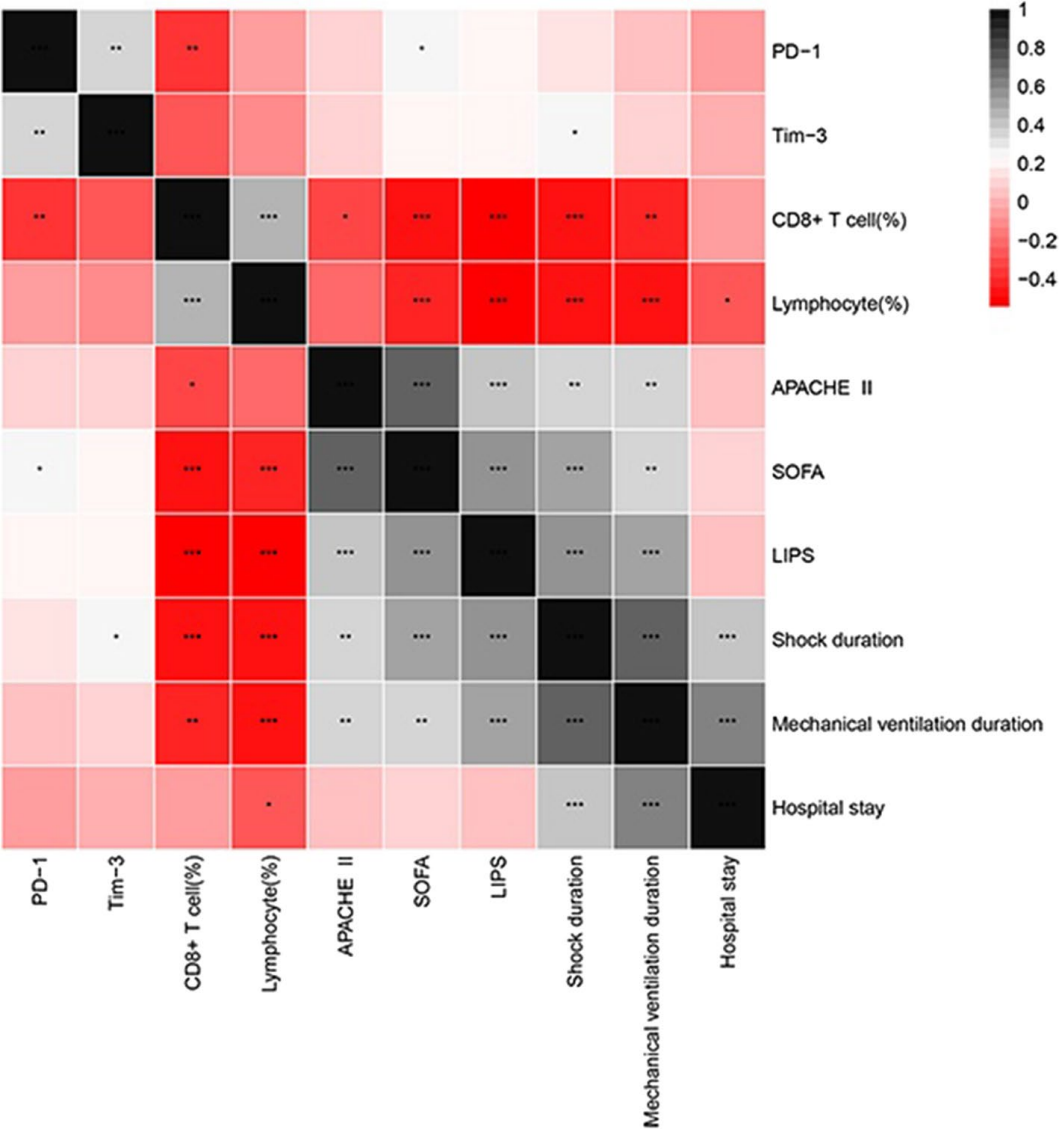
Correlation of CD8^+^ T cells and exhaustion-related molecules with organ dysfunction and progression of ARDS. Correlations between the parameters were analyzed by Spearman’s test. Red represents a positive correlation, and black represents a negative correlation. * p < 0.05, ** *P* < 0.01, ***P < 0.001

Figure 4 shows the results of the Spearman correlation analysis to examine the association of the percentage of lymphocytes, CD8^+^ T cells, and co-signaling molecules with APACHE II score, SOFA score, and lung injury score in ARDS patients. For all patients with ARDS, significant correlations were found between the percentage of lymphocytes and SOFA (ρ = − 0.435, *p* < 0.001) and LIPS scores (ρ = − 0.523, p < 0.001). Additionally, significant associations were also detected between the percentage of CD8^+^ T cells and SOFA (ρ = − 0.457, p < 0.001) and LIPS scores (ρ = − 0.541, p < 0.001). The percentage of PD-1^+^ CD8^+^ T cells was negatively correlated with the percentage of CD8^+^ T cells (ρ = − 0.339, *p* = 0.007) but was positively correlated with the percentage of Tim-3^+^ CD8^+^ T cells (ρ = 0.369, *p* = 0.003) and SOFA score (ρ = 0.267, *p* = 0.036).

Significant associations were detected between the percentage of lymphocytes and CD8^+^ T cells and either shock duration days (ρ = − 0.472, *p* < 0.001 and ρ = − 0.454, p < 0.001) or mechanical ventilation duration days (ρ = − 0.479, p < 0.001 and ρ = − 0.395, *p* = 0.001). The percentage of Tim-3^+^ CD8^+^ cells was positively correlated with shock duration days (ρ = 0.250, *p* < 0.05).

### ROC analyses for predicting 90-day mortality

**Fig. 5:**
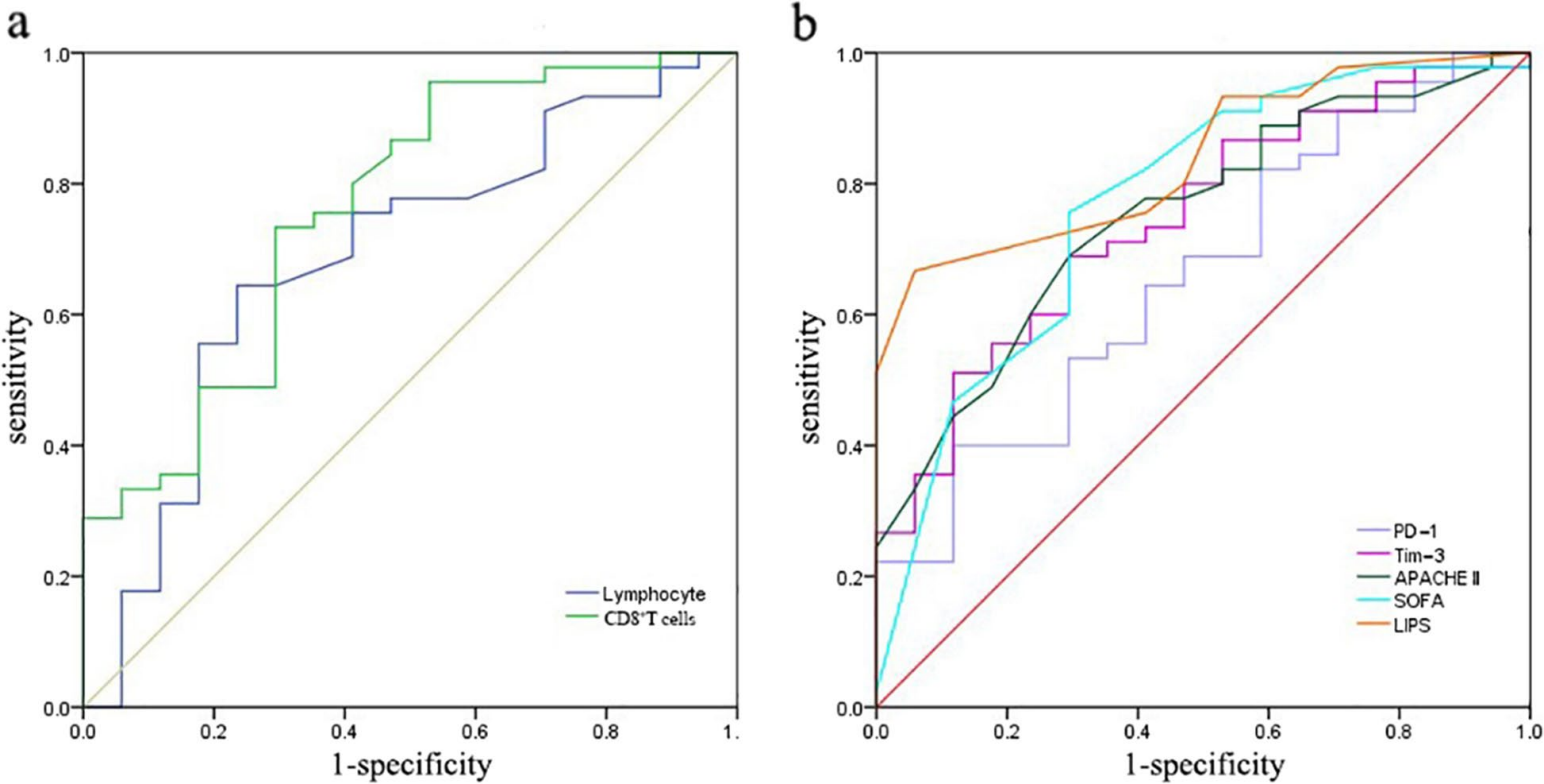
Receiver operating characteristic (ROC) curves for predicting 90-day mortality of ARDS patients. The area under the curve (AUC) demonstrated that the lymphocyte percentage was 0.688 (95% CI 0.535 to 0.842), CD8^+^ T cell percentage was 0.761 (95% CI 0.623-0.898), APACHE II score was 0.752 (95% CI 0.623 to 0.880), SOFA score was 0.770 (95% CI 0.633 to 0.907), LIPS score was 0.839 (95% CI 0.742 to 0.937), PD-1^+^ CD8^+^ T cell percentage was 0.659 (95% CI 0.510 to 0.807) and Tim-3^+^CD8^+^ T cell percentage was 0.745 (95% CI 0.615 to 0.875)

The predictive performance of lymphocytes, CD8^+^ T cells, co-signaling molecules and clinical severity for 90-day mortality in ARDS patients are shown in Fig. 5. The area under the ROC curve (AUC) of the percentage of PD-1^+^ CD8^+^ T cells, Tim-3^+^ CD8^+^ T cells, lymphocytes and CD8^+^ T cells for predicting 90-day mortality were 0.659 (0.510-0.807), 0.745 (0.615-0.875), 0.688 (0.535-0.842) and 0.761 (0.623-0.898), respectively. Moreover, the SOFA score was 0.770 (0.633-0.907), the APACHE II score was 0.752 (0.623-0.880) and the LIPS score was 0.839 (0.742-0.937) (Table 3). The 90-day mortality was predicted according to the cutoff, and the sensitivity and specificity are shown in Table 3.

**Table 3:** The clinical scores and exhaustion-related markers for predicting 90-day mortality

Variables	ROC curve			Sensitivity (%)	Specificity (%)	Youden index (%)
	AUC (95% CI)	Best cutoff	p			
Lymphocytes	0.688(0.535-0.842)	8.75	0.023	64.40	76.50	40.90
CD8^+^ T cell	0.761(0.623-0.898)	6.47	0.002	73.30	70.60	43.90
PD-1	0.659(0.510-0.807)	5.90	0.055	40.00	88.2	28.20
Tim-3	0.745(0.615-0.875)	16.55	0.003	68.90	70.60	39.50
APACHE II Scores	0.752(0.623-0.880)	11.50	0.002	68.90	70.60	39.50
SOFA Scores	0.770(0.633-0.907)	5.50	0.001	75.60	70.60	46.20
LIPS Scores	0.839(0.742-0.937)	7.50	0.000	66.70	94.10	60.80

*Abbreviations:* PD-1, Programmed Cell Death 1; Tim-3, T-cell immunoglobulin mucin-3; APACHE, Acute Physiology and Chronic Evaluation; SOFA, Sequential Organ Failure Assessment; LIPS, Lung Injury Prediction Score; ROC, Receiver operating characteristics; AUC, area under the curve

### Survival curves

**Fig. 6:**
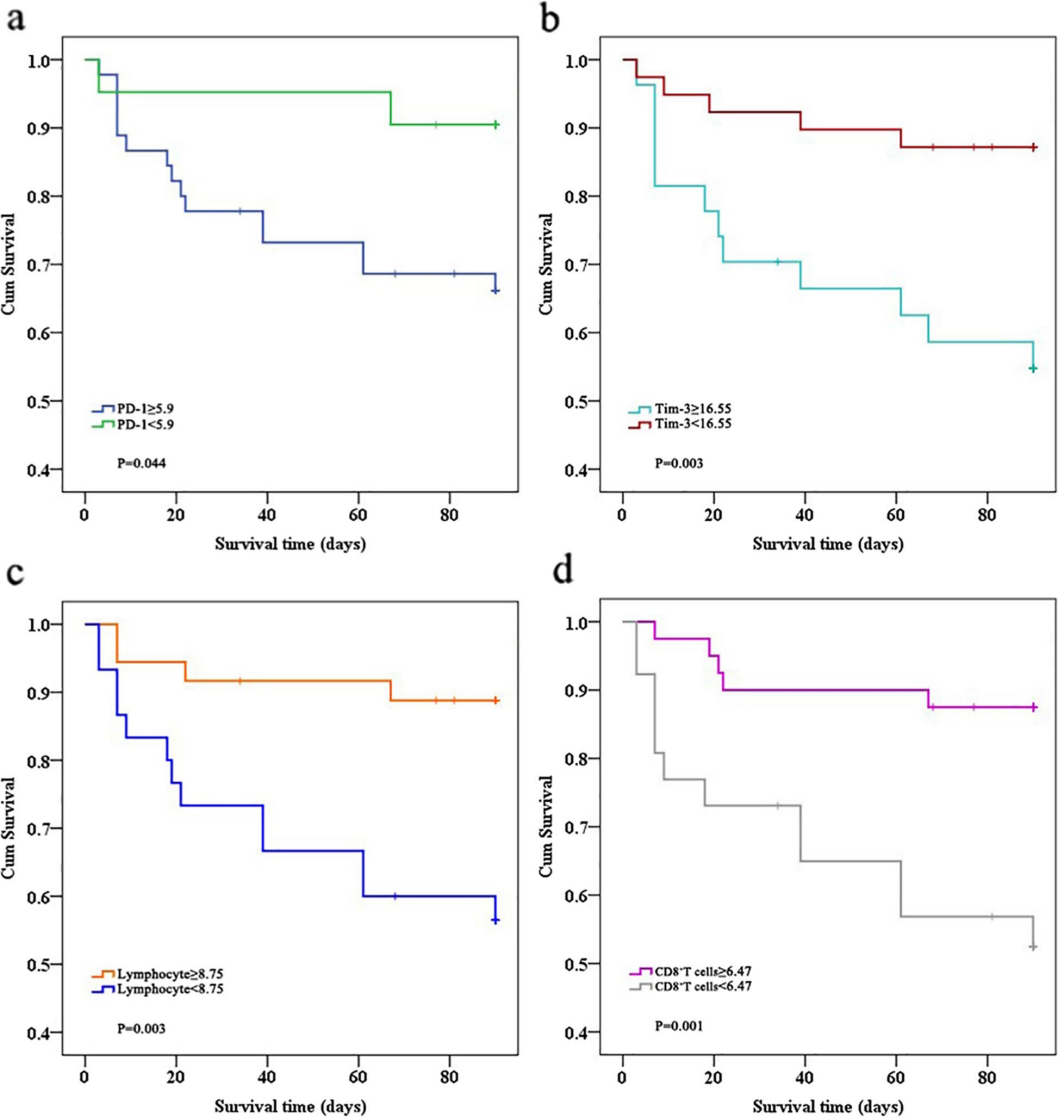
Kaplan–Meier survival curve for patients with ARDS. **a** and **b** Patients with a lower percentage of lymphocytes (< 8.75) and CD8^+^ T cells (< 6.47) had higher mortality at 90 days. **c** and **d** Patients with lower percentages of PD-1^+^CD8^+^ T cells (< 5.9) and Tim-3^+^ CD8^+^ T cells (< 16.55) had a higher probability of survival at 90 days

Survival analysis showed that ARDS patients with a percentage of lymphocytes < 8.75% had higher 90-day mortality (χ2 = 8.317, *p* = 0.004). However, the 90-day mortality rate was lower in ARDS patients with a CD8^+^ T cell ratio > 6.47 (χ2 = 10.698, *p* = 0.001). Patients with a higher percentage of PD-1^+^ CD8^+^ T cells than the cutoff of 5.9% had higher mortality at 90 days (χ2 = 4.193, *p* = 0.041) than patients with a lower percentage of PD-1^+^ CD8^+^ T cells (Fig. 6). The results also showed that the predicted probability of the percentage of Tim-3^+^ CD8^+^ T cells was higher than the cutoff of 16.55%, with higher mortality at 90 days (χ2 = 7.906, *p* = 0.005).

The primary results of this study can be summarized as follows: (1) The counts and percentages of lymphocytes and CD8^+^ T cells in the non-survivor group were lower than those in the survivor group. The proliferation ability of CD8^+^ T cells in the survivor group was greater than that in the non-survivor group. (2) The percentage of PD-1^+^/Tim-3^+^ CD8^+^ cells in ARDS patients who died was significantly higher than that in those who survived. Mortality at 90 days was numerically higher among patients with PD-1^+^ CD8^+^ T cells > 5.9% and Tim-3^+^ CD8^+^ T cells > 16.55%. (3) There was an association between the number of lymphocytes and CD8^+^ T cells and organ failure, especially lung injury. (4) ARDS patients with a higher percentage of PD-1^+^ CD8^+^ T cells showed a significant increase in SOFA scores. The percentage of Tim-3^+^ CD8^+^ T cells was significantly higher in ARDS patients with longer shock durations.

## Discussion

Sepsis-associated ARDS is a condition characterized by bilateral pulmonary infiltrates and inflammation, as well as increased lung epithelial and vascular endothelial permeability [22, 23]. The potential role of lymphocytes in repairing the epithelial and endothelial barrier and evacuating protein-rich edema fluid has been highlighted in recent research based on studies of endotoxin lung injury in mice [9, 24]. There is increasing recognition that a state of impaired immunity follows the initial hyperinflammatory phase [15]. However, few studies have discussed the relationship between CD8^+^ T cell exhaustion and clinical outcomes for patients with sepsis-induced ARDS. This study was conducted to report that CD8^+^ T cells in sepsis may play a role in the prognosis of ARDS induced by sepsis.

Pathogen clearance relies heavily on T cells, with CD8^+^ cytotoxic T cells capable of secreting a variety of molecules such as perforin, granzymes, and IFN-γ to eliminate pathogens from the host [25, 26]. As a result, CD8^+^ T lymphocytes are critical in the clearance of intracellular pathogens and tumors [27]. High and persistent antigen and inflammatory stimulation, on the other hand, usually results in altered CD8^+^ T cell differentiation or exhaustion during chronic infection and malignancies [28–30]. Insufficient levels of circulating lymphocytes may perpetuate a detrimental inflammatory state [31, 32]. Our study found that the counts of CD8^+^ T cells and lymphocytes were significantly reduced in non-survivors compared with survivors, indicating their weakened protective effect on ARDS patients. As is shown in Supplementary material 1, patients with a lower proportion of CD8^+^ T cells and lymphocytes and greater levels of PD-1 and Tim-3 expression had a higher risk of secondary nosocomial infection. Therefore, patients with fewer lymphocytes and CD8^+^ T cells often survived the longer time of shock and mechanical ventilation (Fig. 4). These immune dysfunctions were also indicated in other earlier studies. The study by Dr. Jonathan S et al. revealed that the spleen CD8^+^ T cells of patients who died of sepsis showed exhaustion-like performance [33]. Another study suggested that PD-1 levels in septic shock patients were correlated with increased mortality and nosocomial infection [34].

T cell exhaustion was characterized as the persistence of antigen-specific T cells that had insufficient effector activity, which was originally documented in persistent viral infection [14]. It is distinguished from functional effector or memory T cells by the persistence of inhibitory receptor expression and a weakened proliferative state [35–37]. Previous studies have described that CD8^+^ T cell exhaustion is associated with mortality in various diseases ranging from solid tumors to chronic viral infections and other inflammatory-related diseases [15, 16, 18, 38]. Although they play an important role in other diseases, their role in sepsis-associated ARDS is not well understood [39]. In this study, the proliferation ability of CD8^+^ T cells was significantly decreased in the non-survivors compared to those in survivors. By FACS analysis, we found that CD8^+^ T cells in the non-survivor group had higher levels of both PD-1 and Tim-3 (Fig. 2). In addition, the CD8^+^ T cell co-signaling molecule PD-1 was positively correlated with CD8^+^ T cell counts, suggesting that these cells are undergoing exhaustive changes.

Given the alterations in Tim-3 and PD-1, we need to closely examine their involvement in ARDS. In this study, we discovered that PD-1 and Tim-3 had distinct expression patterns in sepsis-induced ARDS. We analyzed the relationships between the proportion of PD-1^+^/Tim-3^+^ CD8^+^ T cells and relevant clinical indicators. Interestingly, the proportion of PD-1^+^ CD8^+^ T cells was positively correlated with SOFA scores in this study, indicating that the percentage of PD-1^+^ CD8^+^ T cellsmight be able to predict the severity of patients’organ damage. Additionally, the proportion of Tim-3^+^ CD8^+^ T cells was found to be positively associated with the number of days of shock (Fig. 4). This may be because patients with CD8^+^ T cell exhaustion are more likely to develop secondary infections that cause persistent septic shock.

The AUC analysis revealed that the percentage of CD8^+^ T cells and lymphocytes predicted 90-day mortality, similarly to regularly used clinical scoring LIPS, APACHE II, and SOFA scores. ROC analysis revealed that the percentage of CD8^+^ T cells exhibited higher prognostic values than lymphocytes to predict the death of ARDS at 90 days. The cutoff of 6.47% was found to be the optimal value, with a sensitivity of 0.73 and a specificity of 0.71, highlighting the decrease in the number of CD8^+^ T cells may be involved in the immune-mediated worsening condition.. In this study, the inhibitory receptor Tim-3 on CD8^+^ T cells also appeared to be an independent predictor of ARDS, with a sensitivity of 0.69and a specificity of 0.71. Although the predictive sensitivity of PD-1^+^ CD8^+^ T lymphocytes to 90-day mortality is lower than that of Tim-3^+^ CD8^+^ T lymphocytes, their specificity is still 0.88. Therefore, PD-1^+^CD8^+^ T lymphocytes still have certain reference significance for the prediction of mortality. LIPS, APACHE, and SOFA have a propensity to thoroughly evaluate risk factors, vital signs, and biochemical testing to determine the severity of a patient’s disease. Generally speaking, clinical scores always are higher in patients with more severe conditions and worse prognoses [40, 41]. However, CD8^+^ T cell exhaustion plays an important role in evaluating the patient’s immunological state, which is lacking in clinical scores. The common clinical scores are consistent with CD8^+^ T cell exhaustion markers in predicting the prognosis of ARDS. In order to comprehensively assess the patient’s severity, CD8^+^ T cell exhaustion-related markers can be employed as a supplement to the present clinical scores. These findings also encouraged further efforts to explore individualized treatment strategies that target immunological checkpoints PD-1 and Tim-3 and enhance the proliferation of CD8^+^ T cells, which may be able to repair ARDS-induced immunosuppression and protect the host from secondary infections.

However, there was no difference in the cytokines TNF-α and IFN-γ secreted by CD8^+^ T cells between the survivors and the non-survivors. The results were in contrast to earlier studies [42, 43]. This may be due to these cytokines being involved in the exhaustion of CD8^+^ T cells detected in ARDS [25]. Recent studies have shown that TNF-α is a proinflammatory cytokine that can promote T cell apoptosis by interacting with its receptor, TNFR1, which is overexpressed in aged T cells [44, 45]. Our current analysis indicates that TNF-α may be directly involved in promoting T cell exhaustion in these patients. More importantly, these cytokines may also promote the expression of exhaustion markers, including PD-1 and Tim-3, on the surface of peripheral T lymphocytes, leading to reduced T cell function and decreased T cell proliferation [25, 46].

Our study, however, also had limitations that should be addressed. Despite the fact that we had a large number of candidates, the number of patients who were ultimately enrolled in the study was small. Clinical studies with a larger sample size are expected to validate the effect of CD8^+^ T cell exhaustion as soon as possible. In addition, although we discovered that CD8^+^ T cells and surface molecules mediate the prognosis of ARDS, this study, like other studies, was unable to clarify the mechanism of this effect for the reason that the downstream signaling pathway of PD-1 and Tim-3 in ARDS is poorly understood at present [47]. Third, our samples were only collected one time point in this study. Although previous studies revealed that the immunological checkpoints PD-1 and Tim-3 on the cell surface contribute to immunosuppression and are associated with higher mortality, the status of CD8^+^ T cells changes during ARDS [34, 48]. These immunological checkpoints may alter when ARDS patients improve or deteriorate following treatment. The immunological condition over time cannot be explained only by the status of CD8^+^ T cells on admission. Finally, the results of our investigation, which is restricted to ARDS patients with sepsis, cannot be generalized to explain non-sepsis-related ARDS patients since the effects of primary diseases on immune system are inconsistent. Nonetheless, the current study adds to our understanding of CD8^+^ T cell exhaustion in sepsis-induced ARDS.

## Conclusion

In summary, this study suggested that CD8^+^ T cells and co-signaling molecules were promising prognostic markers of sepsis-induced ARDS. Methods for detecting when patients with sepsis-induced ARDS have entered a CD8^+^ T cell exhaustion phase will allow medical staff to detect defects in immunity. Further prospective studies with large samples are needed to confirm these findings and elucidate the underlying mechanism of CD8^+^ T cell exhaustion in sepsis-induced ARDS patients.

## Supplementary Information

**Additional file 1: Supplemental Fig. 1.** Cumulative incidence for secondary infection. a and b Patients with a lower percentage of lymphocytes (< 8.75) and CD8+ T cells (< 6.47) were more likely to develop secondary infection. c and d Patients with lower percentages of PD-1 + CD8+ T cells (< 5.9) and Tim-3+ CD8+ T cells (< 16.55) had a higher probability of secondary infections.

## Data Availability

The datasets generated and/or analyzed during the current study are not publicly available due privacy and ethical restriction but are available from the corresponding author on reasonable request.

## Abbreviations

APACHE: Acute Physiology and Chronic Evaluation
ARDS: Acute respiratory distress syndrome
AUC: Area under the curve
BMI: Body Mass Index
EDTA: Ethylene diamine tetraacetate acid
ICU: Intensive care unit
IFN-γ: Interferon-gamma
LIPS: Lung Injury Prediction Score
PD-1: Programmed Cell Death 1
Tim-3: T-cell immunoglobulin mucin-3
TNF-α: Tumor necrosis factor-alpha
PBMC: Peripheral blood mononuclear cells
ROC: Receiver operating characteristics
SOFA: Sequential Organ Failure Assessment
