# Supplementary material for: Role of CD8+ T cell exhaustion in the progression and prognosis of acute respiratory distress syndrome induced by sepsis: a prospective observational study
Source: BMC Emerg Med. 2022 Nov 19;22:182. doi: 10.1186/s12873-022-00733-2 (PMC9675152; doi:10.1186/s12873-022-00733-2)
Supplement: Supplementary file 1 — Additional file 1: Supplemental Fig. 1. Cumulative incidence for secondary infection. a and b Patients with a lower percentage of lymphocytes (< 8.75) and CD8+ T cells (< 6.47) were more likely to develop secondary infection. c and d Patients with lower percentages of PD-1 + CD8+ T cells (< 5.9) and Tim-3+ CD8+ T cells (< 16.55) had a higher probability of secondary infections. [file 12873_2022_733_MOESM1_ESM.docx]

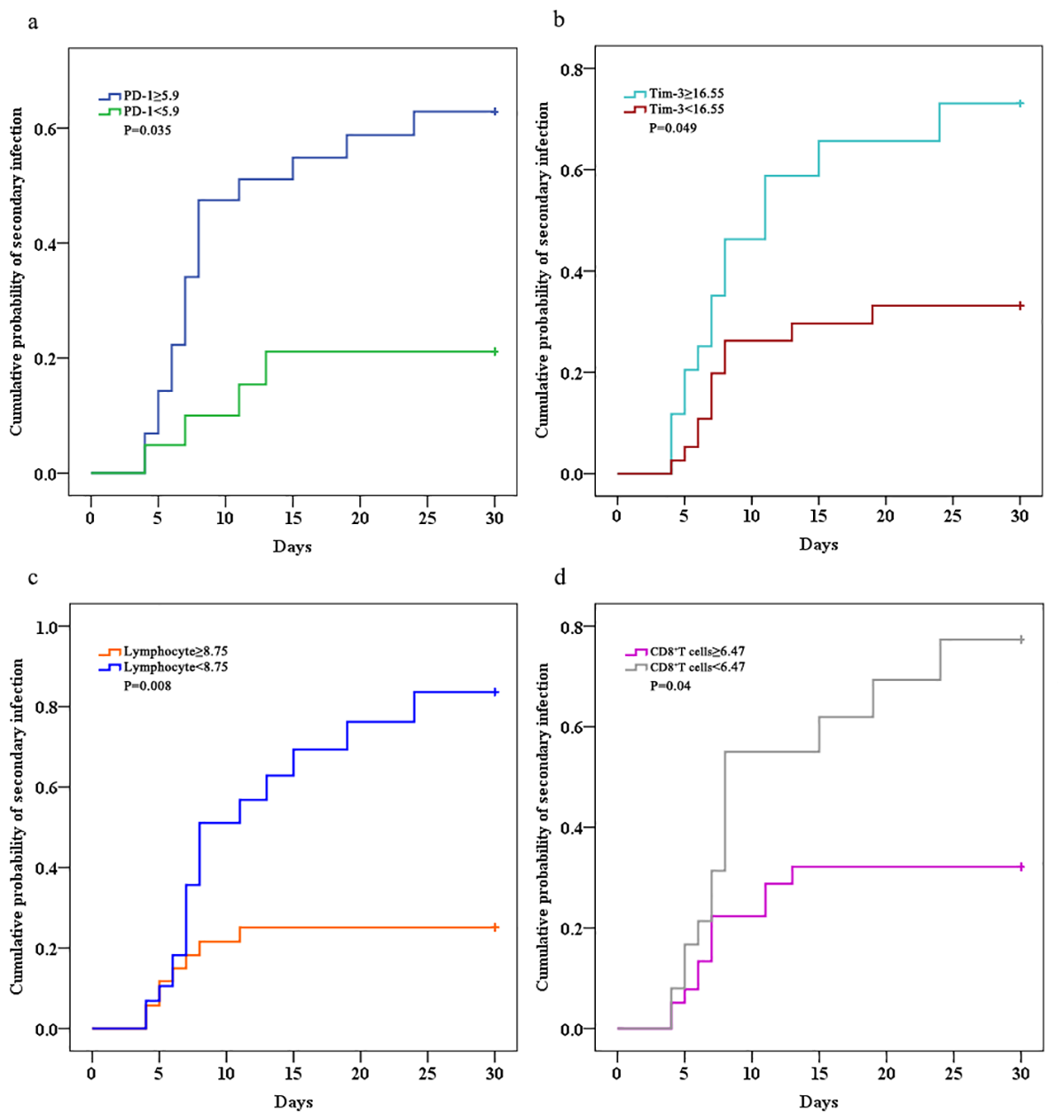


**Supplemental figure 1. Cumulative incidence for secondary infection. a** and **b** Patients with a lower percentage of lymphocytes (<8.75) and CD8^+^ T cells (<6.47) were more likely to develop secondary infection. **c** and **d** Patients with lower percentages of PD-1^+^CD8^+^ T cells (<5.9) and Tim-3^+^ CD8^+^ T cells (<16.55) had a higher probability of secondary infections.
